# Potential Biomarkers for Liver Cancer Diagnosis Based on Multi-Omics Strategy

**DOI:** 10.3389/fonc.2022.822449

**Published:** 2022-02-03

**Authors:** Fanghua Chen, Junming Wang, Yingcheng Wu, Qiang Gao, Shu Zhang

**Affiliations:** ^1^Center for Tumor Diagnosis & Therapy, Jinshan Hospital, Fudan University, Shanghai, China; ^2^Liver Cancer Institute, Zhongshan Hospital, and Key Laboratory of Carcinogenesis and Cancer Invasion (Ministry of Education), Fudan University, Shanghai, China

**Keywords:** hepatocellular carcinoma, multi-omics, biomarker, clinical diagnosis, early detection

## Abstract

Liver cancer is the fourth leading cause of cancer-related death worldwide. Hepatocellular carcinoma (HCC) accounts for about 85%-90% of all primary liver malignancies. However, only 20-30% of HCC patients are eligible for curative therapy mainly due to the lack of early-detection strategies, highlighting the significance of reliable and accurate biomarkers. The integration of multi-omics became an important tool for biomarker screening and unique alterations in tumor-associated genes, transcripts, proteins, post-translational modifications and metabolites have been observed. We here summarized the novel biomarkers for HCC diagnosis based on multi-omics technology as well as the clinical significance of these potential biomarkers in the early detection of HCC.

## Introduction

Liver cancer is one of the leading causes of cancer-related death worldwide (1). Hepatocellular carcinoma (HCC) accounts for > 80% of liver cancer and is usually developed from advanced chronic liver diseases (CLD) with hepatitis virus (mainly HBV and HCV) infection and alcoholic/nonalcoholic liver diseases (2–4). α-fetoprotein (AFP) (5) and Lens culinaris agglutinin-reactive fraction of AFP (AFP-L3) (6, 7), des-gamma-carboxy prothrombin (DCP) (6) and glypican-3 (GPC3) (8, 9) have been used for the clinical diagnosis of HCC. However, the complex pathology and individual heterogeneity of HCC pose great challenges for its early detection (10). Most HCC patients were found at late-stage and had a 5-year survival rate as low as 10.0% (11, 12). It was reported that the 5-year survival rate would be over 86.2% if the patients were given intervention at an early phase (13).

Multi-omics including genomics, epigenomics, transcriptomics, proteomics, glycomics/glycoproteomics and metabolomics can provide novel insights for HCC detection. For genomics and epigenomics, more evidence has shown that circulating tumor DNAs (ctDNAs) and their epigenetic changes could be used as reliable biomarkers (14–16). For transcriptomics, significant changes were observed in mRNAs and noncoding RNAs (miRNAs, lncRNAs, circRNAs) (17). For proteomics, potential protein biomarkers such as Golgi protein-73 (GP73) (18) and heat shock protein 90α (Hsp90α) (19) were identified for HCC detection. For post-translational modifications (PTMs), glycosylation, phosphorylation, acetylation and ubiquitination can be considered for discovering novel biomarkers. Mass spectrometry (MS)-based glycomics/glycoproteomics technology enabled the researchers to characterize aberrant glycoforms and site-specific glycans (20). In addition, metabolomics has contributed to the diagnosis of HCC (21). This review focused on potential biomarkers for liver cancer diagnosis based on multi-omics strategies. Potential HCC biomarkers including genetic mutations, epigenetic changes, mRNAs, noncoding RNAs, proteins, PTMs and metabolites have been summarized in **Table 1**.

**Table 1:** Potential biomarkers for HCC based on multi-omics strategy.

Genes	mRNAs	lncRNAs	miRNAs	circRNAs	Proteins	PTMs	Metabolites
*TP53* (22–29)	*FCN3* (30)	HULC (31)	miR-21 (32)	circ_ZEB1.33 (33)	GP73 (18)	AFP-L3 (Fuc) (6, 7)	1-methyladenosine (34)
*CTNNB1* (c.121A > G, c.133T > C) (22, 35–38)	*CLEC1B* (30)	CYTOR (31, 39)	miR-203 (40)	cSMARCA5 (41, 42)	DCP (5)	A1AT (Fuc) (43)	Xanthine (44)
*TERT* (c.1-124C > T) (22, 36–38)	*PRC1 (*30)	UCA1 (31)	miR-224 (45)	circ_0001445 (46)	AFP (5)	Apo-J (N-glycan) (47)	Uric acid (44)
*AXIN1* (gene mutation) (35, 38)	*YWHAZ* (48)	MALAT1 (31)	miR-20a-5p (49)	circ_000244 (50)	Hsp90α (19)	Fibronectin (Fuc) (51)	Cholyglycine (44)
A 32-gene model (52)	*ENAH* (48)	PTTG3P (31)	miR-25-3p (49)	circ_104075 (53)	OPN (54)	Hemopexin (Fuc) (55)	D-leucic acid (44)
*p16, p15, RASSF1A* (hypermethylation) (56)	*HMGN4* (48)	SPRY4IT1 (31)	miR-30a-5p (49)		MDK (57)	Paraoxonase-1 (Fuc) (58)	3-hydroxy caproic acid (44)
*APC*, *GSTP1*, *RASSF1A*, and *SFRP1* (hypermethylation) (40)	*CAPRIN1* (48)	UBE2CP3 (31)	miR-92a-3p (49)		GPC3 (8, 9)	AGP (Fuc) (59)	Arachidonic lysolecithin (44)
		PTENP1 (31)	miR-132-3p (49)		ANXA2 (60)	Hp (Fuc, Sialic acid) (61, 62)	Dioleoylphosphatidylcholine (44)
		GHET1 (63)	miR-185-5p (49)		ANXA3 (64)	C3, CE, HRG, CD14 (Fuc) (65)	Acetylcarnitine (66)
			miR-320a (49)		DKK1 (67)	4E-BP1 (P) (68)	Butyrylcarnitine (69)
			miR-324-3p (49)		TRX (70)	ALDOA (P) (71)	Hydantoin-5-propionic acid (69)
			miR-375 (49)		PARP1 (72)	ERK1 (P) (73)	Choline (74)
			miR-122 (75)		AFP + fibronectin 1 (76)	ERK2 (P) (73)	Valine (74)
			miR-192 (75)		7-AAb panel (77)	LARP1 (P) (78)	Creatinine (74)
			miR-21 (75)		RNF6 (79)	Smad2/3 (P) (80)	Palmitic acid (81)
			miR-223 (75)		SCCA (70)	Plectin-1 (P) (73)	Phenylalanyl-tryptophan (82)
			miR-26a (75)		CK19 (83)	α-HS-glycoprotein (P) (84)	Glycocholate (82)
			miR-27a (75)			Ku80 (Ub) (85)	
			miR-801 (75)			KLK6 (Ub) (86)	
						SCOS1 (Ub) (87)	
						WDR76 (Ub) (88)	
						AFP (Ac) (89)	
						Core histone H3 (Ac) (90)	
						Core histone H2B (Ac) (91)	
						Core histone H3.3 (Ac) (91)	
						Core histone H4 (Ac) (91)	

*Red represents up-regulation in HCC, blue represents down-regulation in HCC.

TP53, Tumor protein P53; CTNNB1, Catenin beta 1; TERT, Telomerase reverse transcriptase; AXIN1, Axin 1; RASSF1A, Ras association domain family 1 isoform A; APC, Adenomatous polyposis coli; GSTP1, Glutathione S-transferase pi-1; SFRP1, Secreted frizzled-related protein 1; YWHAZ, Tyrosine 3-monooxygenase/tryptophan 5-monooxygenase activation protein zeta; ENAH, Enabled homolog; HMGN4, High mobility group nucleosomal binding domain 4; CAPRIN1, Cell cycle associated protein 1; HULC, Hepatocellular carcinoma upregulated long noncoding RNA; CYTOR, Cytoskeleton regulator RNA; UCA1, Urothelial cancer associated 1; MALAT1, Metastasis associated lung adenocarcinoma transcript 1; PTTG3P, Pituitary tumor-transforming 3 pseudogene; SPRY4-IT1, Sprouty receptor tyrosine kinase signaling antagonist 4 intronic transcript 1; UBE2CP3, Ubiquitin-conjugating enzyme E2C pseudogene 3; PTENP1, Phosphatase and tensin homolog pseudogene 1; GHET1, Gastric carcinoma proliferation enhancing transcript 1; AFP, α-fetoprotein; Hsp90α, Heat shock protein 90α; GP73, Golgi protein 73; DCP, Des-gamma carboxy prothrombin; OPN, Osteopontin; MDK, Midkine; ANXA2, Annexin A2; ANXA3, Annexin A3; DKK1, Dickkopf-1; TRX, Thioredoxin; GPC3, Glypican-3; PARP1, Polymerase 1; 7-AAb panel, CIAPIN1, EGFR, MAS1, SLC44A3, ASAH1, UBL7, and ZNF428; RNF6, Ring finger protein 6; SCCA, Squamous cell carcinoma antigen; CK19, Cytokeratin 19; AFP-L3, Lens culinaris agglutinin-reactive fraction of AFP; A1AT, α-1-antitrypsin; Apo-J, Apolipoprotein J; AGP, α1-acid glycoprotein; Hp, Haptoglobin; C3, Complement C3; CE, Ceruloplasmin; HRG, Histidine-rich glycoprotein; 4E-BP1, 4E-binding protein 1; ALDOA, Aldolase A; ERK1, Extracellular regulated protein kinases 1; ERK2, Extracellular regulated protein kinases 2; LARP1, La-related protein 1; Smad2/3, Mothers against decapentaplegic homolog 2/3; α-HS-glycoprotein, α-Heremans-Schmid-glycoprotein; KLK6, Kallikrein-related peptidase 6; SCOS1, Suppressor of cytokine signaling 1; WDR76, WD40-repeat protein 76; Fuc, Fucosylation; P, Phosphorylation; Ub, Ubiquitination; Ac, Acetylation.

## 1 Genetic Alteration of HCC

Circulating cell-free DNAs (cfDNAs) are DNA fragments released into the peripheral circulation after the degradation of cell components. The increased proliferation and metabolism of tumor cells would release abundant cfDNAs, which could be used as biomarkers (92–96). The conventional genetic changes of HCC, including point mutations, microsatellite changes and chromosomal rearrangements are reflected in cfDNAs. It was reported that about 50 somatic alterations had been detected and then they could cause changes of their corresponding proteins in HCC (97, 98). Among 48 patients, 56.3% of patients had at least one mutation of the four sites in the following three genes (c.747 for *TP53*, c.121, c.133 for *CTNNB1* and c.1-124 for *TRET*), which could be further found in 22.2% of HCC patients’ tissues (22, 23). Moreover, the R249S mutation in *TP53* was proved to have a potential diagnostic value in the test of 895 HCC patients (24). Other mutation sites of *TP53*, such as 157 (25), 175 (26), 245 (27), 248 (28) and 273 (29) have been considered for HCC detection. In addition, amino acid changes, such as S37 and S33, could be used as available monitoring indicators for HCC (35–37). The combination of cfDNA mutations and protein changes can increase the diagnosis accuracy of HCC. For example, HCC diagnosis using *TP53*, *TERT*, *CTNNB1*, AFP and DCP has achieved satisfactory results (38).

Epigenetic alterations of ctDNAs were associated with HCC. Methylation changes of ctDNAs often occur in the early stage of tumorigenesis, particularly those alterations in the CpG islands of anti-oncogenes, which may play critical roles in the initiation and progression of HCC (99). The 5-hydroxymethylcytosines (5hmC) are abundantly expressed epigenetic markers (100). A 32-gene diagnostic model was developed using the 5hmC-Seal technique, which accurately distinguished early-stage HCC from non-HCC (52). Accumulating studies have also reported the aberrant methylation of glutathione S-transferase pi-1 (*GSTP1*) promoter region and cyclin-dependent kinase inhibitor *p15* and *p16* in HCC patients (101–103). Moreover, the combination of several hot methylated genes was utilized for HCC diagnosis. For example, *p16*, *p15* and ras association domain family 1A (*RASSF1A*) were assessed in 50 HCC patients and they provided an overall predictive accuracy of 89% with a sensitivity of 84% and a specificity of 94% (56). A panel of four genes (*APC*, *GSTP1*, *RASSF1A* and *SFRP1*) could make a distinction between HCC and normal controls with a sensitivity of 92.7% and a specificity of 81.9% (104). A predictive model that consisted of three abnormally methylated genes (*APC*, *COX2* and *RASSF1A*) and one miRNA (miR-203) could be considered to diagnose HCC (40, 105).

## 2 Transcriptomics of HCC

Analysis of differential gene expression was important for HCC detection. Three genes (*FCN3*, *CLEC1B* and *PRC1*) were explored to be HCC biomarkers based on large-scale transcriptome datasets (30). It was found that *YWHAZ*, *ENAH*, *HMGN4* and *CAPIRN1* changed significantly in HCC (48). Transcriptomics can be integrated into other omics for biomarkers screening. A transcriptome-proteome assay was performed to track the possible biomarkers from HCC-derived gene expression to its protein product released into serum, and a candidate biomarker, Hsp90α, was identified (106).

Long noncoding RNAs (lncRNAs), microRNAs (miRNAs) and circular RNAs (circRNAs) play important roles in the epigenetic regulation of gene expression (107–109). Especially, lncRNAs can affect the expression and stability of miRNAs and messenger RNAs (mRNAs) (110–112). It was reported that lncRNAs HULC and CYTOR were used for joint diagnosis of HCC (39). Moreover, a diagnostic panel including lncRNAs CYTOR, UCA1 and AFP had satisfactory sensitivity and specificity (31).

miRNAs are small noncoding RNAs composed of 20-24 nucleotides. Elevated miR-21 levels in HCC patients have been widely reported (32). The high expression of miR-224 had a good diagnostic value with an AUC of 0.888-0.899 for patients with early HCC (45). It was reported that the overexpression of eight miRNAs (miR-20a-5p, miR-25-3p, miR-30a-5p, miR-92a-3p, miR-132-3p, miR-185-5p, miR-320a and miR-324-3p) had diagnostic significance in HBV positive HCC patients. Besides, the four miRNAs (miR-20a-5p, miR-320a, miR-324-3p and miR-375) panel could contribute to the early screening of HCC (49). Zhou et al. found plasma miR-122, miR-192, miR-21, miR-223, miR-26a, miR-27a and miR-801 were potential circulating biomarkers for HCC (75). This seven-miRNA panel showed high accuracy in the diagnosis of HCC, even in patients with early stages. The miRNA panel enabled a more detailed distinction among HCC, healthy, chronic HBV and cirrhosis. It has been evaluated by multi-center clinical detection and authorized by National Medical Products Administration in China.

The changes in circRNA expression were also observed in HCC patients (113). For example, cSMARCA5 (41, 42), circ-ZEB1.33 (33) and circ_0001445 (46) levels were notably decreased in HCC patients. Two circRNAs (circ_000244 and circ_104075) were upregulated in HCC tissues and sera. The circ_104075 exhibited better sensitivity and specificity than some traditional HCC biomarkers (53). The circ_000244 showed better diagnostic performance than AFP (50).

## 3 Proteomics of HCC

Many classical proteins, which served as reliable biomarkers for other cancers, have been posing new value in HCC diagnosis. Squamous cell carcinoma antigen (SCCA) was previously reported to be associated with cervical cancer (114). Recent studies also indicated that it had a significant contribution to the early diagnosis of HCC (70). Protein expression of cytokeratin 19 (CK19) in HCC was low, however, it would reflect the malignant progression of hepatoma cells (83). GP73 (18), osteopontin (OPN) (54), midkine (MDK) (57), annexin A2 (ANXA2) (60), annexin A3 (ANXA3) (64), dickkopf-1 (DKK1) (67), thioredoxin (TRX) (70) and polymerase 1 (PARP1) (72) have shown diagnostic value for the early diagnosis of liver cancer. Ring finger protein 6 (RNF6) was upregulated and promoted the tumorigenicity of HCC, which might be useful for the detection of HCC at the initial stage (79). A combination of protein markers was also considered for HCC detection, for example, the joint diagnosis with AFP and fibronectin 1 (76). A novel 7-autoantibody (AAb) panel containing CIAPIN1, EGFR, MAS1, SLC44A3, ASAH1, UBL7 and ZNF428 was identified using HCC-focused array (77). The artificial neural network model was established for this panel and it was also able to detect AFP-negative HCC with AUC values of 0.841-0.948. Further, proteogenomics was used to address the complex biological properties of cancer and it could incorporate proteomics into genomic-level studies to obtain more accurate cancer information (115).

## 4 Post-Translational Modifications (PTMs) of HCC

**Figure 1:**
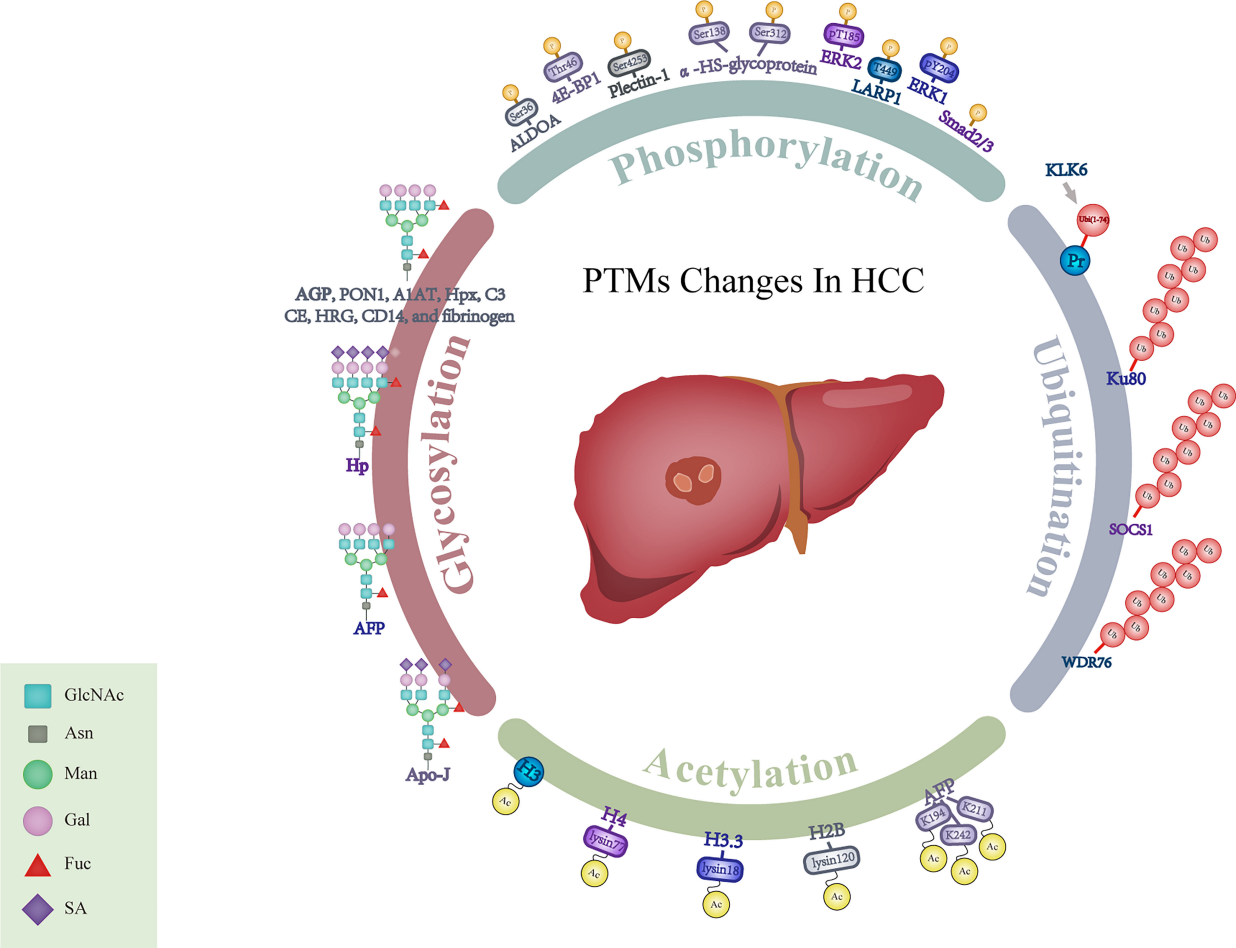
Schematic representation for alterations of PTMs in HCC. GlcNAc, N-acetylglucosamine; Asn, Asparagine; Man, Mannose; Gal, Galactose; Fuc, Fucose; SA, Sialic Acid; AGP, α1-acid glycoprotein; PON1, Paraoxonase 1; A1AT, α-1- antitrypsin; Hpx, Hemopexin; C3, Complement C3; CE, Ceruloplasmin; HRG, Histidine-rich glycoprotein; Hp, Haptoglobin; AFP, α-fetoprotein; Apo-J, Apolipoprotein J; ALDOA, Aldolase A; 4E-BP1, 4E-binding protein 1; ERK1, Extracellular regulated protein kinases 1; ERK2, Extracellular regulated protein kinases 2; LARP1, La-related protein 1; Smad2/3, Mothers against decapentaplegic homolog 2/3; α-HS-glycoprotein, α-Heremans-Schmid-glycoprotein; KLK6, Kallikrein-related peptidase 6; Pr, Protein; SOCS1, Suppressor of cytokine signaling 1; WDR76, WD40-repeat protein 76; H2B, Histone 2B; H3.3, Histone 3.3; H4, Histone 4; H3, Histone 3.

PTMs such as glycosylation, phosphorylation, acetylation and ubiquitination play vital roles in multiple physiological processes and disease progression, including control of the cell cycle progression, changes of chromatin structures and transduction of cellular signals (116). We have summarized potential biomarkers with different PTMs in **Figure 1**.

### Glycosylation of HCC

Tremendous evidence illustrated that glycan structures were altered in cancers (117, 118). Cancer-associated glycosylation aberration provides novel biomarkers by utilizing glycomic/glycoproteomic technologies (119–123). For example, the glycan profile has been assessed to predict the development of HCC in cirrhosis (124). Glycomics is to detect glycans attached to macromolecules such as proteins (7, 119, 121, 125) and glycoproteomics is a high-throughput technique that could reveal glycosylation sites and site-specific glycoforms (126).

Different glycosylation patterns mainly occur in fucosylation, glycan branching, sialylation and terminal N-acetylgalactosamine (127–129). It was reported that increased fucosylated N-glycans played crucial roles in cancer development, such as core-α-1,6-fucosylated triantennary glycan (130–132). Fucosylation of α1-acid glycoprotein (AGP) was increased in patients with liver cirrhosis and HCC. Meanwhile, different degrees of fucosylation could further distinguish HCC from liver cirrhosis. Thus, determining the specific changes of AGP glycan structures could be helpful for HCC detection (59). The combination of trifucosylated N-glycan of AGP, AFP and AGP showed superiority in discriminating HCC from liver cirrhosis. Zhu et al. investigated the alterations in fucosylation degree of serum haptoglobin (Hp) in a cohort, which included healthy controls, liver cirrhosis and HCC patients, and also confirmed that the fucosylation abnormalities of Hp were closely related to HCC (62). The monofucosylated triantennary glycan at Asn184 and Asn241 of Hp had the diagnostic potential for HCC patients (133). In addition, enhanced fucosylation of serum paraoxonase 1 (PON1) (58), α-1-antitrypsin (A1AT) (43), hemopexin (Hpx) (55), complement C3 (C3), ceruloplasmin (CE), histidine-rich glycoprotein (HRG), CD14 (65) and fibrinogen (51) have been reported to be potential glycobiomarkers for early-stage HCC detection.

High-mannose levels were reported to be associated with HCC (134, 135). Previous studies have shown that N-glycosylation changes occurred in the progression of HCC (136). A total of 83 N-glycans was identified in HCC, and among them, 57 had alterations (137). Two glycopeptides of IgA_2_ might be unique glycan signatures and provided diagnostic clues in HBV-related liver cancer (138). Apolipoprotein J (Apo-J) in HCC had decreased levels of triantennary glycan and the level of glycosylation of Apo-J could differentiate HCC from cirrhosis with an AUC of 0.852 (47). Besides, sialylation played an important role in cell recognition, adhesion and signal transduction. The high content of sialic acid has been observed in HCC (139). Three glycans containing sialic acid have been regarded as candidate markers for the detection of HCC and they could differentiate HCC from CLD with the AUC of 0.89-0.93 (140). The sialylated glycans of serum Hp were also elevated in HCC (61, 141).

### Phosphorylation of HCC

Aberrant protein phosphorylation is associated with HCC (142) and more phosphorylation alterations have been elucidated with the development of omics methods. Elevating phosphorylation levels of 4E-binding protein 1 (4E-BP1) on Thr46 could be used to predict the early recurrence and metastasis of HCC (68). The level of Ser36 phosphorylation of aldolase A (ALDOA) was increased and could be used as a potential biomarker for HCC (71). Changes of some phosphorylation sites, such as the remarkable downregulation of pT185 on extracellular regulated protein kinases 2 (ERK2) and pY204 on extracellular regulated protein kinases 1 (ERK1), have contributed to the progression of HCC (73). Phosphorylation of plectin-1 (phospho-Ser-4253) and α-HS-glycoprotein (phospho-Ser 138 and 312) were also found to be potential HCC biomarkers (84). Furthermore, phosphorylation of la-related protein 1 (LARP1)-T449 and mothers against decapentaplegic homolog 2/3 (Smad2/3)-Thr8 could be useful for HCC detection (78, 80).

### Ubiquitination of HCC

Ubiquitination modification, mediating protein enzymatic degradation by labeling proteins, plays a critical role in tumorigenesis (143). The change of Lys48-linked ubiquitination in heterogeneous nuclear ribonucleoprotein A1 (HNRNPA1) inhibited HNRNPA1-dependent pyruvate kinase isozyme splicing, and subsequently promoted glucose metabolism reprogramming and malignant behavior of cells in HCC (144). Kallikrein-related peptidase 6 (KLK6) and its catalytic products Ubi1-74 could identify cirrhotic patients at risk of developing HCC (86). The ubiquitination levels of several proteins could serve as HCC indicators, such as lupus Ku autoantigen protein p80 (Ku80) (85), suppressor of cytokine signaling 1 (SCOX1) (87) and WD40-repeat protein 76 (WDR76) (88).

### Acetylation of HCC

Acetylation modification, as a dynamic and particular component of PTMs, has attracted more attention in recent years. Lysine acetylation is regulated by the interaction between acetylase and deacetylase (145). Increasing evidence has shown that lysine acetylation played a pivotal role in metabolic function and cellular signaling transduction in the occurrence and development of HCC (63, 146). The sites of lysine acetylation in non-histone proteins and histone proteins have been studied in liver tissues (147, 148). Using MS detection, the acetylation at K194, K211 and K242 of AFP provided novel markers and therapeutic targets for HCC (89). Additionally, the acetylation levels of lysine 120 in histone H2B, lysine 18 in histone H3.3 and lysine 77 in histone H4 were found to be increased in HCC (91). Core histone H3 is another highly conserved protein in cell nucleus and its acetylation has indicated diagnostic significance in HCC (90).

## 5 Metabolomics of HCC

Metabolites with low molecular weight such as < 1.5 kDa can be defined as “metabolome”, and these small molecular metabolites can dynamically change in liver diseases (149). Metabolomics is a high-throughput method to identify and measure metabolites and offers an opportunity to discover biomarkers (150). Many metabolites were identified including xanthine, uric acid, cholyglycine, D-leucic acid, 3-hydroxy caproic acid, arachidonic acid lysolecithin and dioleoylphosphatidylcholine. They could be effective for the discrimination of HCC from HCV (44). Serum acetylcarnitine enabled clinicians to detect HCC from liver cirrhosis (66). In addition, palmitic acid made a distinction between liver cirrhosis and HBV. The 5-methoxytryptamine, malic acid and phenylalanine were used to discriminate HBV and normal controls. The β-glutamate and asparagine were potential liver disease-specific biomarkers to distinguish HCC from liver cirrhosis (74). Serum 1-methyladenosine was identified as a characteristic metabolite for HCC (34). Two metabolites, butyrylcarnitine and hydantoin-5-propionic acid could be combined together to detect HCC (69). A total of 169 genes and 28 metabolites was reported to be associated with HCC (81). The product of stearoyl CoA desaturase, monounsaturated palmitic acid, increased the invasiveness of HCC, enhanced the migration ability of HCC cells *in vitro* and might be helpful for HCC diagnosis.

## Discussion

Omics technologies and analytical software have been improved. For glycosylation, pGlyco (151), StrucGP (152), GPQuest and GlycoPAT (153) helped to obtain detailed and accurate data. Multi-omics proposed more biomarkers and the specificity and sensitivity of these biomarkers still need to be comprehensively evaluated. Previous studies showed that the profile of DNA methylation had high tissue specificity and helped to determine the tissue origin of cfDNAs (154–157). The combination of different omics biomarkers and the application of computational models can increase diagnostic accuracy. For example, monitoring the change of HCC-specific CpG island methylator phenotype in company with AFP was proved to have better diagnostic performance (158). Measuring both Mac-2 binding protein glycosylation isomer (M2BPGi) and AFP improved the detection sensitivity (159). Metabolites such as phenylalanyl-tryptophan and glycocholate could be added to the traditional HCC diagnostic process to achieve early detection (82).

Different types of biomarkers and detection methods have their advantages and applicable fields. The changes of some proteins and ctDNAs can be detected in the early stages of cancer and they may have high sensitivity for detecting high-risk patients. Considering the affinity of lectin and glycan-specific antibodies to their corresponding glycosylated structures may be low, so its detection usually needs more complex methods (160, 161). Milliliters of plasma were often used for cfDNA extraction (162); micrograms of proteins or microliters of serum seem to be enough for PTM determination (163); for metabolites, microliters of serum were often considered (164). Thus, different methods need to be considered and improved to promote clinic application of multi-omics biomarkers.

## Conclusion

The combination of multi-omics, including genomics, transcriptomics, proteomics, glycomics, glycoproteomics and metabolomics would provide more sensitive and accurate detection for HCC, especially in the early stage. Multi-omics approaches also enable the researchers to gain deeper insight into the molecular mechanism of HCC development. With optimized technologies and clinical validation, multi-omics biomarkers would become practical in clinic for HCC diagnosis.

## Author Contributions

FC and SZ collected information and wrote the manuscript. JW made the table and drew the figure. YW made modifications to the manuscript. QG and SZ formulated the writing frame and made important modifications to the manuscript. All authors contributed to the article and approved the submitted version.

## Funding

The work was supported by the Science and Technology Commission of Shanghai Municipality (20JC1418900) and Shanghai Pujiang Program (2020PJD012).

## Conflict of Interest

The authors declare that the research was conducted in the absence of any commercial or financial relationships that could be construed as a potential conflict of interest.

## Publisher’s Note

All claims expressed in this article are solely those of the authors and do not necessarily represent those of their affiliated organizations, or those of the publisher, the editors and the reviewers. Any product that may be evaluated in this article, or claim that may be made by its manufacturer, is not guaranteed or endorsed by the publisher.
